# Expanded unbiased population-genomic summary statistics in pixy

**DOI:** 10.64898/2026.07.27.741083

**Published:** 2026-07-30

**Authors:** Kieran Samuk, Matt Stone, Erin McAuley, Nick P. Bailey, Gina Lucas, Mikhail Plaza

## Abstract

Estimates of population-genetic summary statistics are often computed from variant-only VCFs, which commonly omit *invariant* sites (i.e. sites with only homozygous reference genotypes across all samples). However, these sites are critical for correctly estimating per site statistics such as nucleotide diversity (π) and between population divergence (d _xy_ ). Our software pixy addressed this issue by providing support for computing statistics directly from “all-sites” VCFs that encode invariant positions explicitly (Korunes and Samuk 2021). pixy has since been widely adopted and used in a large variety of population genetic studies. Here we present a major update to pixy, which expands the original tool in four major areas. First, we provide implementations of new estimators of Watterson’s θ and Tajima’s D that are unbiased with respect to missing data. Secondly, we provide support for arbitrary ploidy and multiallelic sites. Third, we introduce a variety of optimizations, including multicore execution and a roughly order-of-magnitude reduction in per-worker memory footprint. Finally, we have broadly modernized our code base, test suite, and community contribution pathway. We validate these new features on simulated polyploid and multiallelic datasets, as well as two empirical datasets (one diploid and one autotetraploid), and benchmark the new multicore scaling. We position pixy relative to contemporary tools and discuss shared limitations of VCF-based estimators. By integrating support for diverse ploidies, allelic complexity, and genome-wide scale in one workflow, pixy provides the population genetics community with a straightforward tool for estimates of key summary statistics that are unbiased with respect to missing data. pixy remains completely open-source (MIT-licensed) and easily installable with the conda package management system.

## Full Text Availability

The license terms selected by the author(s) for this preprint version do not permit archiving in PMC. The full text is available from the preprint server.

